# Mapping of the QTLs governing grain nutrients in wheat (*Triticum aestivum* L.) under nitrogen treatment using high-density SNP markers

**DOI:** 10.3389/fpls.2025.1553525

**Published:** 2025-05-12

**Authors:** Animireddy China Malakondaiah, Sudhir Kumar, Hari Krishna, Biswabiplab Singh, Sukumar Taria, Monika Dalal, R. Dhandapani, Lekshmy Sathee, Renu Pandey, Ranjeet Ranjan Kumar, Viswanathan Chinnusamy

**Affiliations:** ^1^ Division of Plant Physiology, Indian Council of Agricultural Research (ICAR)-Indian Agricultural Research Institute, New Delhi, India; ^2^ Division of Genetics, Indian Council of Agricultural Research (ICAR)-Indian Agricultural Research Institute, New Delhi, India; ^3^ Division of Biotechnology, Indian Council of Agricultural Research (ICAR)-National Institute for Plant Biotechnology, New Delhi, India; ^4^ Division of Biochemistry, Indian Council of Agricultural Research (ICAR)-Indian Agricultural Research Institute, New Delhi, India

**Keywords:** recombinant inbred lines, nitrogen-deficient, multi-trait genotype ideotypes distance index, quantitative trait locus, logarithm of odds, grain zinc concentration

## Abstract

**Introduction:**

Micronutrient deficiencies, particularly zinc (Zn) and iron (Fe), are prevalent global health issues, especially among children, that lead to hidden hunger. Wheat is a primary food source for billions of people, but it contains low essential minerals. According to recent studies, the optimum application of nitrogen (N) fertilizers can significantly enhance the micronutrient uptake and accumulation in wheat grains.

**Methods:**

The aims of this study were to identify superior wheat recombinant inbred lines (RILs) of RAJ3765 × HD2329 with high nutrients in grain using the multi-trait genotype–ideotype distance index (MGIDI) and to identify quantitative trait loci (QTLs)/genes associated with grain nutrient content using a single-nucleotide polymorphism (SNP)-based genetic linkage map. The parents and their RIL population were grown under control and nitrogen-deficient (NT) conditions, and nutrient content was determined using inductively coupled plasma optical emission spectroscopy (ICP-OES).

**Results and discussion:**

Analysis of variance and descriptive statistics showed a significant difference among all the nutrients. The highest mean values of grain iron concentration (GFeC) and grain zinc concentration (GZnC) were 52.729 and 35.137 mg/kg, respectively, under the control condition, while the lowest mean values were 41.016 and 33.117 mg/kg, respectively, recorded under NT; a similar trend was observed in all the elements. Genotyping was carried out using the 35K Axiom^®^ Wheat Breeder’s Array. A genetic linkage map was constructed using 2,499 polymorphic markers identified for parents across 21 wheat chromosomes. Genetic linkage mapping identified a total of 26 QTLs on 17 different chromosomes. A total of 18 QTLs under the control condition and eight QTLs under the nitrogen stress condition were identified. QTLs for each nutrient were selected based on the high percentage of phenotypic variation explained (PVE%) and logarithm of odds (LOD) score value of more than 3. The LOD scores for studied nutrients varied from 3.04 to 13.42, explaining approximately 1.1% to 27.83% of PVE. One QTL was mapped for grain calcium concentration (GCaC), whereas two QTLs each for grain potassium concentration (GKC), GFeC, grain copper concentration (GCuC), and grain nickel concentration (GNiC) were mapped on different chromosomes. Four QTLs were mapped each for GZnC, grain manganese concentration (GMnC), and grain molybdenum concentration (GMoC), while the highest five were linked to grain barium concentration (GBaC). *In silico* analysis of these chromosomal regions identified putative candidate genes that code for 30 different types of proteins, which play roles in many important biochemical or physiological processes. Putative candidate gene magnesium transporter MRS2-G linked to GFeC and probable histone-arginine methyltransferase CARM1 and ABC transporter C family were found to be linked to GZnC. These QTLs can be utilized to generate cultivars adapted to climate change by marker-assisted gene/QTL transfer.

## Introduction

Wheat (*Triticum aestivum* L.) is one of the most extensively cultivated crops worldwide and comprises nearly 20% of calories and almost 40% of protein globally (Abdelrahman et al., 2020). Wheat is the major staple grain of approximately 2.5 billion consumers across 89 countries in the world (Wheat Summary Report, CGIAR, 2017-2021). Hidden hunger is a term used to describe the abnormally high rate of malnutrition among children caused by micronutrient deficiencies (Harding et al., 2018; Godecke et al., 2018). Approximately 27% of children under five in Africa and 69% of children in Asia suffered malnutrition or illness in 2017 (https://www.who.int/nutgr owthd b/2018-jmebroch ure.pdf). It has been shown that malnutrition as a result of micronutrient (mostly zinc and iron) deficiency affects a large percentage of the global population. Furthermore, according to a report given by the World Health Organization, globally, more than 2 billion people suffer from a deficiency of zinc and iron (Bhutta and Salam, 2013). To maintain food security, India must double the production of food by 2050 (Ray et al., 2013). Chhuneja et al. (2006) found that *Aegilops tauschii* is a rich source of high micronutrient content and contains more grain Zn and Fe contents than agricultural wheat. Nitrogen (N) fertilizer is the primary input for crop production, and thus, to double the food production, a significantly higher amount of approximately 73% nitrogenous fertilizers is required for wheat production (FAO, 2005). However, the continued application of a single nutrient disrupts the nutritional balance and leads to the depletion of other nutrients. The single-nutrient approach has often presented multiple nutrient deficiencies in wheat crops. The distribution of nitrogen fertilizer dosages and the method of application in plant vegetation are crucial to improving fertilizer use efficiency and increasing both the quantity and the quality of cereal grains (Wojtkowiak et al., 2014). The lack of coordination between the addition of nitrogen to the soil and the needs of plants is one of the main causes of low nitrogen usage efficiency in practice (Cassman et al., 2002; Fageria and Baligar, 2005). Nitrogen fertilizer has been shown to alter the micronutrient concentration in cereal grains (Cakmak et al., 2010; Shi et al., 2010). According to recent research, nitrate or ammonium-related nitrogen fertilization can increase the accumulation of iron, zinc, and copper in wheat grains. It can encourage the accumulation of these elements in cereal grains (Kutman et al., 2010). Several articles have indicated that the status of grain micronutrients may be impacted by the management of nitrogen fertilizers, even though the effect of nitrogen fertilization on micronutrient density has seldom been investigated. For instance, Morgounov et al. (2007) discovered a substantial positive correlation between protein content, Zn, and Fe. According to Shi et al. (2010), a high N application raised the concentrations of Zn and Cu in all three milling fractions and the concentration of Fe in bran and shorts but not in flour. Micronutrients are crucial for improving productivity because they regulate the synthesis of proteins, chlorophyll, and nucleic acids (Rehm and Albert, 2006). They also actively participate in several enzymatic processes involved in photosynthesis and respiration (Reddy et al., 2004). Grain protein content decreases when zinc levels are low (Brown et al., 1993).

The genetic basis for micronutrient aggregation in grains and quantitative trait locus (QTL) mapping needs to be understood for increasing grain micronutrient concentration. Tiwari et al. (2009) reported two QTLs for Fe in grain on chromosomes 2A and 7A and one QTL for grain Zn on 7A. In addition, the most important locus regulating the concentration of Cu in grain was the chromosomal region on chromosome 2A linked to Xgwm95. In a durum wheat × wild emmer wheat recombinant inbred line (RIL) population, it was shown to have grain Fe, Zn, Cu, and Mg contents (Peleg et al., 2009). Cakmak et al. (1999) found that the D genome of wheat contributes to the high zinc efficiency, but it is considered to be the least genetically diverse. Furthermore, four QTLs for the Zn content were mapped on chromosome 7A, which explains the highest phenotypic variance (Shi et al., 2008), and one QTL on chromosomes 3D (Xu et al., 2012) 3A, 5A, 7A, 4B, 7B, and 5D (Manjunath et al., 2023). Four QTLs for grain iron (GFe) and seven QTLs for grain zinc (GZn) were identified by Wang et al. (2021), some of which showed pleiotropic effects on both traits. QTLs mapped for Fe concentration on chromosomes 2B (Yasmin et al., 2014), 1D, 2D, 4B, 4D 5B, 6B, and 6D (Manjunath et al., 2023). Krishnappa et al. (2022) reported five Marker trait associations (MTAs) for Fe on chromosomes 1A, 5A, 6A, 3B, and 7B, explaining the phenotypic variance of 12.7%–24.1% and also for Zn on chromosomes 6A, 2B, 5B, and 7B, explaining the phenotypic variance of 5.7%–10.9%. Four significant MTAs were identified for grain iron concentration (GFeC) on chromosomes 2B, 3A, 3B, and 6A, and two were identified on chromosomes 1A and 7B for grain zinc concentration (GZnC) (Rathan et al., 2022). Additionally, a panel of 768 wheat cultivars was genotyped using genome wide association study (GWAS) analysis of 45,298 single-nucleotide polymorphisms (SNPs) from 55K chip arrays, and 154 QTLs were found for eight traits across three environments. Three stable QTLs (qMn-3B.1, qFe-3B.4, and qSe-3B.1/qFe-3B.6) were identified across different environments or traits (Hao et al., 2024).

Therefore, to prevent hidden hunger and enhance the wheat crop productivity per unit area, improving the quality of wheat grains from normal (control) and treatment (nitrogen-deficient) conditions is most important. This study reflects the effect of available nitrogen on grain nutrient levels. It was carried out in greenhouses of climate-controlled Nanaji Deshmukh Plant Phenomics Facility, ICAR-IARI, New Delhi. Phenomics is the multidisciplinary study of high-throughput, precise acquisition, and analysis of multidimensional phenotypes using digital sensors to record the morphological and physiological responses of plants (Kumar et al., 2016). The main objective of this study was to estimate the nutrient concentration and to identify QTLs/genes linked with nutrient traits in wheat grains utilizing genetic linkage mapping under control and nitrogen treatment in parents and their RILs.

## Materials and methods

### Plant material and experimental design

The mapping population comprised 81 recombinant inbred lines (RILs) and their parents, namely, RAJ3765 × HD2329. Plants of RILs and parents were grown in plastic pots filled with 12 kg soil. The soil N status was measured before the sowing of wheat. Plants were cultivated under open air conditions until anthesis, followed by controlled environments (temperature: day, 20°C ± 2°C; night, 14°C ± 2°C; 40% humidity). The RIL population and parents were assessed under two treatments, namely, recommended fertilizer dose (120:80:60 kg/ha N:P:K, respectively) and recommended fertilizer dose minus nitrogen (0:80:60 kg/ha N:P:K, respectively), and taken up for this research. The design of the experiment was completely randomized design (CRD), with two replications, and six plants per treatment were used for every RIL and parent. This experiment was conducted in the climate-controlled greenhouse of Nanaji Deshmukh Plant Phenomics Facility, ICAR-IARI, New Delhi.

### Phenotyping for grain micronutrients

Grain samples were taken at the physiological maturity (PM) stage for the nutrient content estimation. The grain nutrient content was determined utilizing inductively coupled plasma optical emission spectrometry (ICP-OES Agilent 5110). The nutrient content was estimated by digesting the samples with di-acid digestion (Paul et al., 2017). Ten milliliters of digestion solution (a mixture of nitric and perchloric acid in a ratio of 9:4 v/v) was added into 1 g of the sample and then kept overnight for pre-digestion. The flasks or digestion tubes were heated slowly (at approximately 100°C) and then heated at a higher temperature (approximately 260°C or more) until the solution became translucent. When the organic material was fully digested, a brownish smoke was released. Until the volume was decreased to 3 to 5 mL, but not completely dry, the content was evaporated. After that, the flasks were allowed to cool to room temperature. Quantitatively, the digested materials were moved into 50-mL volumetric flasks. Ash-free quantitative filter paper (Whatman No. 41, Whatman International Ltd., Kent, UK) was used to complete the transfer (https://www.epa.gov/sites/default/files/2015-12/documents/3050b.pdf). Aliquots of this solution were used to determine micronutrients using the ICP-OES Agilent 5110. ICP-OES can be used to detect up to 19 elements in one analytical procedure. The instrument was warmed up with argon gas for 30 minutes to create active plasma containing Ar+ ions; 5% of HNO_3_ (5 mL con. HNO_3_ + 95 mL of double-distilled water) was always used as a blank. All the standards were prepared in 5% HNO_3_. The standard solution of more than 30 days old was not used because of changes in elemental concentrations with age. It works by exciting atoms in a sample with plasma energy. When these excited atoms return to their ground state, they emit light at specific wavelengths. By measuring the intensity of the emitted light, the concentration of each element can be determined. A series of standard solutions with known and increasing concentrations of the analyte were prepared (Charles and Fredeen, 1997). The signal intensity was measured for each standard. The signal intensity was plotted against the corresponding concentration to create a calibration curve. Unknown sample concentrations were determined by comparing their signal intensities to the calibration curve (Ghosh et al., 2013).

### Phenotypic data analysis

Descriptive statistics for minimum, maximum, mean values, standard deviation (SD), and coefficient of variation (CV) were calculated using JASP (https://jasp-stats.org/). One-way and two-way analyses of variance (ANOVAs) were calculated utilizing the linear model (lm) function in the R software. In addition, in the two-way ANOVA, the effect size was calculated using the “lsr” packages in the R programming. The confidence interval was calculated using 95% significance levels. Principal component analysis was conducted utilizing the R software package “FactoMineR package ver. 2.4” (Husson et al., 2016), and the graphical visualization of principal component analysis (PCA) outcomes was carried out using the R package “factoextra version 1.0.7” (Kassambara and Mundt, 2020). Scaled data were used for PCA.

### Genotyping and QTL analysis

Leaf samples collected from the RILs and parent population in 21-day-old seedlings were used for DNA extraction using the cetyltrimethylammonium bromide (CTAB) method (Murray and Thompson, 1980). Using λ DNA as the standard and 0.8% agarose gel electrophoresis, the quality of the genomic DNA was assessed and measured using NanoDrop. Genotyping all the RILs with parents was conducted utilizing 35K SNP-based markers with a commercial high-density Affymetrix Axiom genotyping array/Wheat Breeders’ Array (Allen et al., 2017). Using SNP markers (Singh, 2023) and the IciMapping (Li et al., 2007; Meng et al., 2015) 4.2 software/R software, the linkage map of the RIL population was developed. The Kosambi mapping tool was used to calculate the map distances between markers. QTL analysis was carried out utilizing inclusive composite interval mapping (ICIM) with the IciMapping ver. 4.2 software (Wang, 2009). The best linear unbiased prediction (BLUP) values of phenotypic and genotypic variance were utilized for QTL discovery. Using the “Deletion” command, missing phenotypic data were removed. All QTLs had a walking speed of 1.0 cM, and stepwise regression showed p = 0.001. For the identification of putative QTLs, a logarithm of odds (LOD) threshold of 3.0 with 1,000 permutations was selected. The standard nomenclature found in the wheat gene symbol library was used to label the identified QTLs (McIntosh et al., 2013). The locations of the QTL’s flanking markers were utilized to identify the potential genes. To find potential candidate genes in the physical locations of markers against the IWGSC wheat (*T. aestivum* L.) reference genome integrated into the Ensembl Plants database, a search using the Basic Local Alignment Search Tool (BLAST) was conducted. Furthermore, using the BioMart tool in the Ensembl Plants platform, potential candidate genes were identified based on the positions of flanking markers of the respective QTLs. BLASTP at the National Center for Biotechnology Information (NCBI) was also used to confirm candidate genes that code for proteins.

### Identification of superior genotypes using MGIDI

The multi-trait genotype–ideotype distance index (MGIDI) was utilized to select superior RILs based on information related to multiple traits (Olivoto and Nardino, 2021). The MGIDI was determined using the following formula:


$$\text{MGIDIi}=\sqrt{\sum_{j=1}^{f}{\left(F_{ij}-F_{j}\right)}^{2}}$$

where F_ij_ is the ith genotype’s score in the jth factor (i = 1, 2, …, g; j = 1, 2, …, f); g and f are the numbers of RILs and factors, respectively; F_j_ is the jth score of the ideotype. The MGIDI for the ith genotype is represented by MGIDI i. The genotype with the least MGIDI is more closely aligned with the ideotype; it should exhibit the desired traits for all the traits that have been examined. For the ideotype plan, all the nutrients were assigned as higher values, and MGIDI calculation was carried out in the R software ver. 4.2.2 utilizing the “metan” package (https://tiagoolivoto.github.io/metan/reference/Smith_Hazel.html.).

## Results

### Phenotypic variability

The one-way ANOVA revealed that there was a highly significant variation in all the studied nutrient contents in the mapping population under both control and nitrogen treatment (NT). One-way ANOVA, effect size, and confidence interval of nutrient content in grains under control and NT are given in **Supplementary Tables S1A** and **B**. The two-way ANOVA results of the population indicated that there was significant variance in genotype, conditions, and genotype vs. conditions (G vs. E) interaction for all the nutrients. The maximum effect size of the genotype was observed in grain molybdenum concentration (GMoC) (0.701), while the lowest was recorded in grain barium concentration (GBaC) (0.331); the effect size of treatment, G vs. E, and confidence interval of nutrients are mentioned in **Tables 1A** and **B**. The phenotypic variability of different nutrients with their minimum, maximum, mean values, SD, and CV were calculated in RILs (**Table 2**) and their parents under control and NT conditions (**Supplementary Tables S2A, B**). Frequency distribution plots showed that all nutrients were distributed normally under both conditions. Distribution plots utilizing histograms showing different nutrients under both the studied conditions are given in **Supplementary Figure S1**.

**Table 1A:** Two-way ANOVA for GCaC, GKC, GMgC, GFeC, GZnC, and GMnC in wheat grains.

Source of variations	Degree of freedom	GCaC	GKC	GMgC	GFeC	GZnC	GMnC
GEN	81	15,590***	278,962***	45,989***	240.9***	90.33***	21.14***
ENV	1	90,913****	1,729,281***	342,718***	11,261.9***	338.40***	23.194*
GEN : ENV	80	7,152	186,955***	16,506	57.3***	47.48***	11.506***
Residuals	161	7,122	79,707	13,085	6.4	26.42	5.268
Effect size (GEN)		0.411	0.434	0.497	0.535	0.464	0.488
Effect size (TRT)		0.029	0.033	0.045	0.309	0.021	0.006
Effect size (GEN : TRT)		0.186	0.286	0.176	0.125	0.241	0.262
Conf. interval (95%)		579.317–600.637	2,878.639–2,966.443	806.057–839.356	45.743–48.063	33.322–34.846	12.314–13.034

GCaC, grain calcium concentration; GKC, grain potassium concentration; GMgC, grain magnesium concentration; GFeC, grain iron concentration; GZnC, grain zinc concentration; GMnC, grain manganese concentration.

*** and * indicate 0.1% and 5% significance levels, respectively.

**Table 1B:** Two-way ANOVA for GMoC, GCuC, GAlC, GNaC, GBaC, GNiC, and GSrC in wheat grains.

Source of variations	Degree of freedom	GMoC	GCuC	GAlC	GNaC	GBaC	GNiC	GSrC
GEN	81	0.23756***	4.873***	1.608***	20,903***	1.998***	0.014563***	2.7942***
ENV	1	0.26651***	17.91**	17.846***	147,301***	5.247*	0.018401**	2.1605*
GEN : ENV	80	0.06169***	3.241**	0.966***	3,734	1.915***	0.004929***	0.9388***
Residuals	161	0.01861	2.046	0.429	2973	1.041	0.002482	0.4779
Effect size (GEN)		0.701	0.394	0.433	0.647	0.331	0.591	0.594
Effect size (TRT)		0.009	0.017	0.060	0.056	0.010	0.009	0.005
Effect size (GEN : TRT)		0.179	0.258	0.262	0.114	0.313	0.197	0.197
Conf. interval (95%)		0.395–0.459	3.384–3.769	2.325–2.534	263.203–282.883	1.576–1.844	0.188–0.205	3.785–4.022

GMoC, grain molybdenum concentration; GCuC, grain copper concentration; GAlC, grain aluminum concentration; GNaC, grain sodium concentration; GBaC, grain barium concentration; GNiC, grain nickel concentration; GSrC, grain strontium concentration.

***, **, and * indicate 0.1%, 1%, and 5% significance levels, respectively.

**Table 2:** Grain elemental concentration (mg/kg) for recombinant inbred lines (RILs) in control (C) and nitrogen-deficiency (T) conditions.

Trait	Treatment	Mean	Std. error of mean	SD	CV	Minimum	Maximum
GCaC	C	606.674	8.670	78.030	0.129	453.470	772.665
	T	573.705	7.951	71.556	0.125	401.920	717.835
GKC	C	2,994.949	39.245	353.201	0.118	2,371.315	3,995.010
	T	2,845.899	37.469	337.219	0.118	2,088.200	3,671.890
GMgC	C	854.879	14.757	132.816	0.155	544.350	1,129.255
	T	789.435	13.190	118.714	0.150	503.195	1,079.960
GFeC	C	52.729	1.092	9.824	0.186	29.470	71.535
	T	41.016	0.790	7.109	0.173	22.370	56.860
GZnC	C	35.137	0.627	5.644	0.161	25.435	50.075
	T	33.117	0.685	6.167	0.186	18.860	47.655
GMnC	C	13.029	0.317	2.849	0.219	6.625	19.095
	T	12.408	0.282	2.538	0.205	6.345	17.570
GMoC	C	0.451	0.030	0.272	0.602	0.085	1.060
	T	0.393	0.028	0.249	0.634	0.040	0.920
GCuC	C	3.688	0.133	1.197	0.324	1.335	6.110
	T	3.347	0.125	1.124	0.336	1.115	6.165
GAlC	C	2.663	0.093	0.840	0.315	1.310	4.670
	T	2.195	0.085	0.768	0.350	0.895	4.125
GNaC	C	294.272	7.396	66.563	0.226	128.650	451.675
	T	251.518	9.981	89.831	0.357	79.440	428.710
GBaC	C	1.754	0.094	0.842	0.480	0.375	3.910
	T	1.473	0.071	0.639	0.434	0.265	2.915
GNiC	C	0.205	0.008	0.071	0.348	0.030	0.390
	T	0.189	0.008	0.069	0.365	0.040	0.350
GSrC	C	3.983	0.091	0.817	0.205	2.430	6.060
	T	3.775	0.109	0.982	0.260	2.000	5.854

SD, standard deviation; CV, coefficient of variation; GCaC, grain calcium concentration; GKC, grain potassium concentration; GMgC, grain magnesium concentration; GFeC, grain iron concentration; GZnC, grain zinc concentration; GMnC, grain manganese concentration; GMoC, grain molybdenum concentration; GCuC, grain copper concentration; GAlC, grain aluminum concentration; GNaC, grain sodium concentration; GBaC, grain barium concentration; GNiC, grain nickel concentration; GSrC, grain strontium concentration.

**Figure 1:**
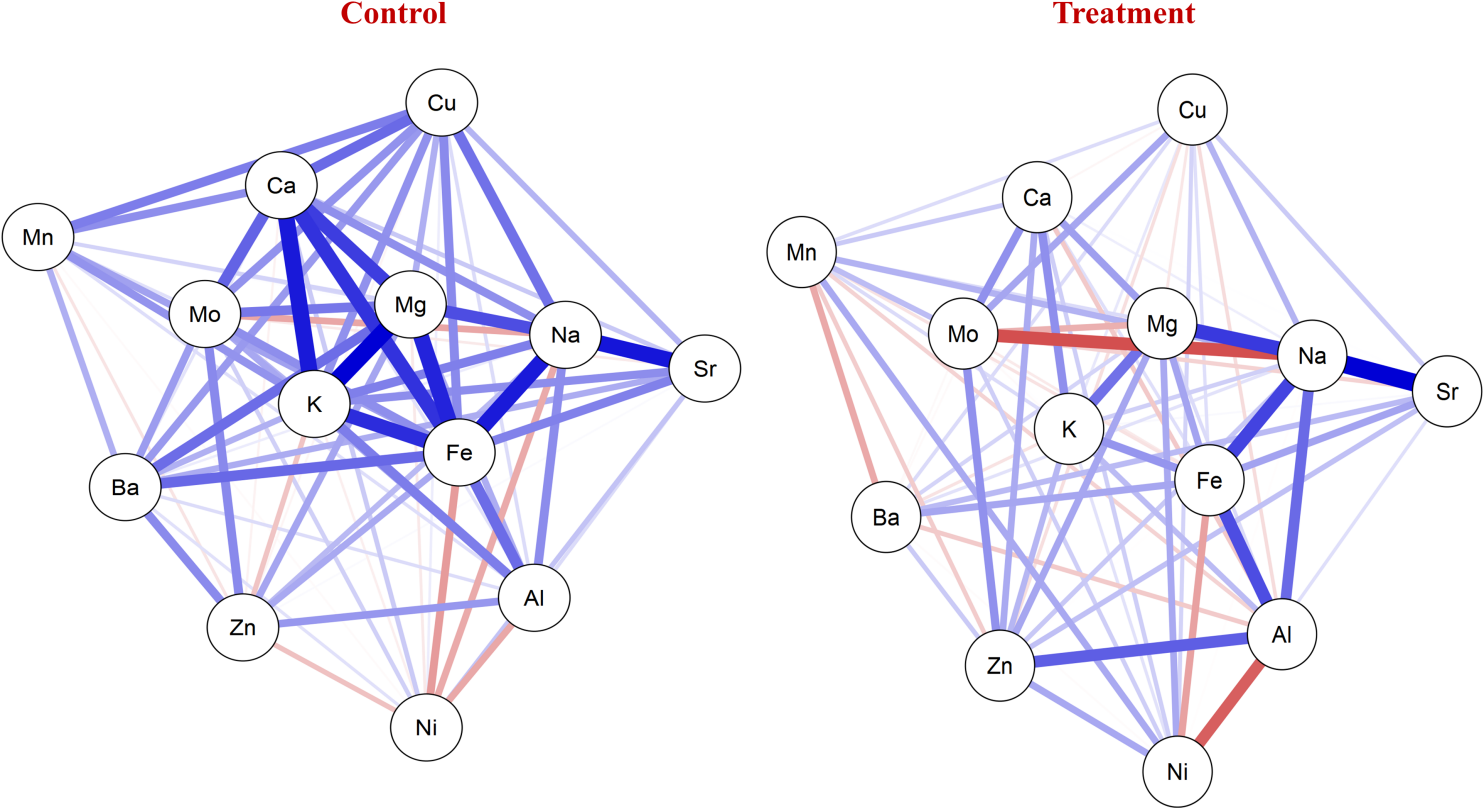
Network plot showing correlation of nutrients under control and nitrogen deficiency (NT). Blue lines represent positive correlation between nutrients. Red lines represent negative correlation among nutrients.

The mean values of all the nutrients were the highest under control, while the lowest values were recorded under the NT condition. The mean values under the control condition for: grain calcium concentration (GCaC), grain potassium concentration (GKC), grain magnesium concentration (GMgC), GFeC, GZnC, grain manganese concentration (GMnC), GMoC, grain copper concentration (GCuC), grain aluminum concentration (GAlC), grain sodium concentration (GNaC), GBaC, grain nickel concentration (GNiC), and grain strontium concentration (GSrC) were 606.674, 2,994.949, 854.879, 52.729, 35.137, 13.029, 0.451, 3.688, 2.663, 294.272, 1.754, 0.205, and 3.983 mg/kg, respectively. In contrast, under the NT condition, their concentrations were 573.705, 2,845.899, 789.435, 41.016, 33.117, 12.408, 0.393, 3.347, 2.195, 251.518, 1.473, 0.189, and 3.775 mg/kg, respectively (**Table 2**). The network correlation plot under the control condition revealed strong positive correlations between nutrient pairs such as GNaC–GSrC, GFeC–GMgC, GKC–GCaC, and GFeC–GCaC. Conversely, negative correlations were observed between GMoC–GNaC, GFeC–GAlC, and GAlC–GNiC. Under the NT condition, positive associations were noted among GFeC–GAlC, GNaC–GSrC, and GFeC–GMgC, whereas negative correlations were found for GMoC–GNaC, GFeC–GAlC, and GFeC–GNiC (**Figure 1**).

### Principal component analysis

**Figure 2:**
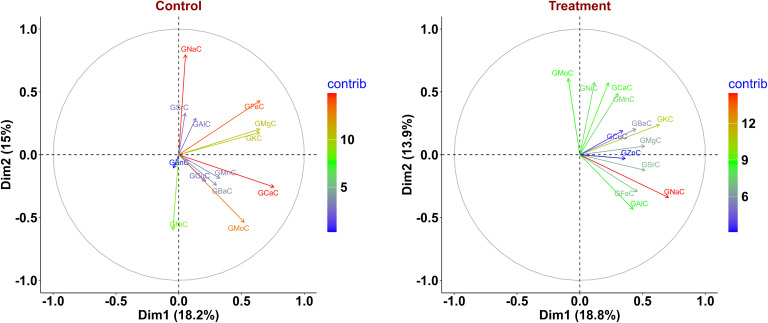
Principal component analysis (PCA) biplot displaying variance in phenotype of wheat recombinant inbred line (RIL) population under control and nitrogen-deficiency (NT) conditions. GCaC, grain calcium concentration; GKC, grain potassium concentration; GFeC, grain iron concentration; GMgC, grain magnesium concentration; GMnC, grain manganese concentration; GMoC, grain molybdenum concentration; GZnC, grain zinc concentration; GNiC, grain nickel concentration; GCuC, grain copper concentration; GNaC, grain sodium concentration; GAlC, grain aluminum concentration; GBaC, grain barium concentration; GSrC, grain strontium concentration.

Principal component analysis under the control condition revealed that the first four principal components contributed 55.56% of the cumulative variation. Dimension-1 (Dim-1) contributed 18.2% of the overall variation, while Dim-2, contributed 15% of the cumulative variance (**Figure 2**). For Dim-1, the major contributors were GCaC, GFeC, GMgC, and GKC, while GNaC, GNiC, GMoC, and GFeC contributed to Dim-2. Similarly, nutrients like GZnC, GAlC, and GMnC contributed to Dim-3 and Dim-4; the major contributors were GSrC, GBaC, GCuC, and GKC (**Supplementary Figure S2A**). The nutrients GFeC, GMgC, GKC, GMnC, and GCuC were grouped together with acute angles (<90°), indicating a positive association between them. GNiC showed a negative association with GFeC and GNaC with acute angles (>90°) among them (**Figure 2**).

Under the NT condition, PCA indicated that the first four principal components contributed 52.37% of the cumulative variation. Dim-1 contributed 18.8% of the total variance, whereas Dim-2 contributed 13.9% of the cumulative variance (**Figure 2**). Traits like GNaC, GKC, GSrC, and GMgC contributed to Dim-1, and for Dim-2, the major contributors were GMoC, GNiC, and GCaC. Furthermore, traits like GSrC, GAlC, GNiC, and GMgC contributed to Dim-3, whereas GZnC and GFeC contributed to Dim-4 (**Supplementary Figure S2B**). Acute angles between traits GZnC, GFeC, GCuC, GSrC, and GMgC showed a positive association across them. Similarly, GMoC, GFeC, and GNiC did not cluster together at acute angles (>90°), suggesting a negative association between them (**Figure 2**). The eigenvalues, variance percentage, and overall variance under both environments are given in **Supplementary Table S3**.

### QTL and *in silico* analysis for grain nutrients

**Figure 3:**
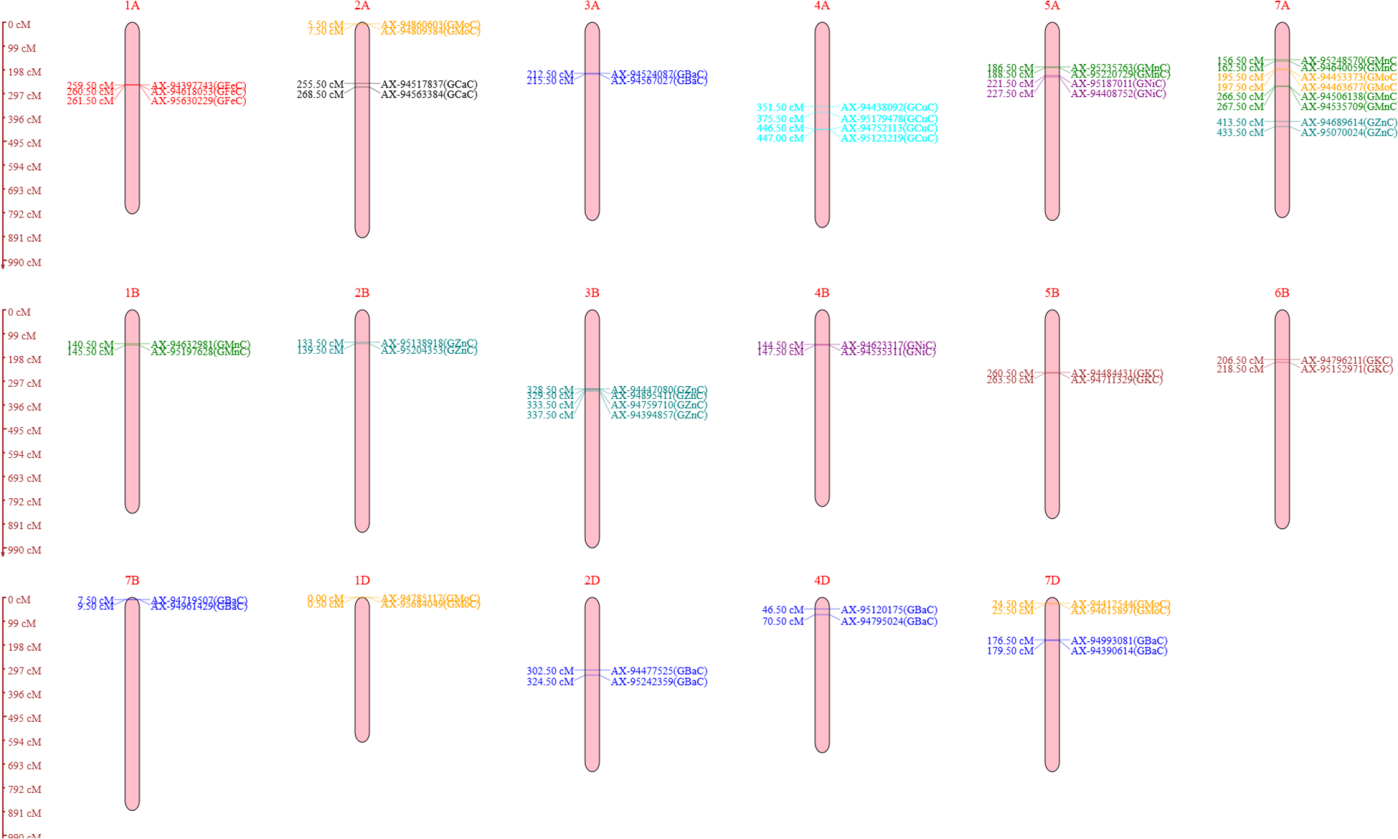
Genomic regions occupied by quantitative trait loci (QTLs) selected with logarithm of odds (LOD) score value >3 (all the identified QTLs on 17 different chromosomes) for the analyzed nutrients under both control and nitrogen-deficiency (NT) conditions. Black color represents QTLs for grain calcium concentration (GCaC). Brown color represents QTLs for grain potassium concentration (GKC). Red color represents QTLs for grain iron concentration (GFeC). Green color represents QTLs for grain manganese concentration (GMnC). Orange color represents QTLs for grain molybdenum concentration (GMoC). Teal color represents QTLs for grain zinc concentration (GZnC). Purple color represents QTLs for grain nickel concentration (GNiC). Cyan color represents QTLs for grain copper concentration (GCuC). Blue color represents QTLs for grain barium concentration (GBaC).

A genetic linkage map was generated utilizing 2,499 polymorphic markers identified for RAJ3765 and HD2329 across 21 wheat chromosomes. The R software was used to create the linkage map of the RIL population utilizing SNP markers. ICIM investigation resulted in a total of 26 QTLs identified on 17 different chromosomes. Out of 26 QTLs, 18 were found under the control condition, which were associated with GCaC, GKC, GFeC, GZnC, GMnC, GMoC, GBaC, and GNiC. In the case of the NT condition, eight QTLs identified were linked to GKC, GZnC, GMnC, GMoC, GCuC, and GBaC. QTLs for each nutrient were selected based on a LOD score value of more than 3 and a high percentage of phenotypic variance explained (PVE%). The LOD score of studied nutrients ranged from 3.04 to 13.42, which explains approximately 1.1% to 27.83% of PVE. One QTL was mapped for GCaC, whereas two QTLs were mapped each for GKC, GFeC, GCuC, and GNiC. Four QTLs were mapped each for GZnC, GMnC, and GMoC, and the highest of five were linked to GBaC. The additive effect was negative for 15 QTLs, and a positive additive effect was observed in 11 QTLs, illustrating that the favorable alleles for these loci were inherited from parent RAJ3765 or HD2329 (**Table 3**). The QTLs were selected with a LOD score >3.0, which were located on 17 different chromosomes highlighted with various colors (**Figure 3**).

**Table 3:** Significant quantitative trait loci (QTLs) containing logarithm of odds (LOD) score value of more than 3 for nutrients under control (C) and nitrogen-deficient (T) condition and also phenotypic variation explained (PVE%).

Trait	Condition	QTL name	Chr	Position	Left marker	Right marker	LOD	PVE (%)	Add	LeftCI	RightCI
GCaC	C	QGCaC.iari-2A	2A	268	AX-94517837	AX-94563384	3.245	14.1331	−30.6906	255.5	268.5
GKC	C	QGKC.iari-5B	5B	261	AX-94484431	AX-94711329	3.53	10.9941	134.7959	260.5	263.5
GKC	T	QGKC.iari-6B	6B	209	AX-94796211	AX-95152971	3.8917	13.7002	−86.7771	206.5	218.5
GFeC	C	QGFeC.iari-1A.1	1A	259	AX-95630456	AX-94397743	9.3115	6.0637	6.0053	258.5	259.5
GFeC	C	QGFeC.iari-1A.2	1A	261	AX-94618053	AX-95630229	13.4239	10.1023	−7.7575	260.5	261.5
GZnC	C	QGZnC.iari-2B	2B	139	AX-95138918	AX-95204353	6.6603	10.9652	2.9988	133.5	139.5
GZnC	C	QGZnC.iari-3B.1	3B	337	AX-94759710	AX-94394857	3.5645	5.2972	−2.0778	333.5	337.5
GZnC	C	QGZnC.iari-7A	7A	425	AX-94689614	AX-95070024	5.5429	15.1395	3.5224	413.5	433.5
GZnC	T	QGZnC.iari-3B.2	3B	329	AX-94447080	AX-94895411	3.2596	10.0593	−1.9184	328.5	329.5
GMnC	C	QGMnC.iari-1B	1B	145	AX-94632981	AX-95197628	3.0451	1.109	−0.8196	140.5	145.5
GMnC	C	QGMnC.iari-7A.1	7A	267	AX-94506138	AX-94535709	3.1721	27.835	−4.1172	266.5	267.5
GMnC	T	QGMnC.iari-5A	5A	188	AX-95235763	AX-95220729	3.5648	14.6053	−0.9306	186.5	188.5
GMnC	T	QGMnC.iari-7A.2	7A	160	AX-95248570	AX-94640059	3.2359	12.5249	−0.8305	156.5	162.5
GMoC	C	QGMoC.iari-1D	1D	0	AX-94785117	AX-95684049	4.9777	9.8753	0.0807	0	0.5
GMoC	C	QGMoC.iari-2A	2A	7	AX-94860603	AX-94809384	4.9671	10.1139	0.0823	5.5	7.5
GMoC	C	QGMoC.iari-7A	7A	197	AX-94453373	AX-94463677	6.7507	14.7439	−0.0981	195.5	197.5
GMoC	T	QGMoC.iari-7D	7D	25	AX-94412544	AX-94615897	3.4566	9.2758	0.0768	24.5	25.5
GCuC	T	QGCuC.iari-4A.1	4A	363	AX-94438092	AX-95179478	3.3119	17.7586	−0.5661	351.5	375.5
GCuC	T	QGCuC.iari-4A.2	4A	447	AX-94752113	AX-95123219	3.4103	7.2581	0.3728	446.5	447
GBaC	C	QGBaC.iari-2D	2D	324	AX-94477525	AX-95242359	4.4612	12.7819	−0.3672	302.5	324.5
GBaC	C	QGBaC.iari-4D	4D	69	AX-95120175	AX-94795024	3.2092	8.8961	0.3058	46.5	70.5
GBaC	C	QGBaC.iari-7B	7B	9	AX-94719507	AX-94961429	3.0731	8.0111	−0.2899	7.5	9.5
GBaC	C	QGBaC.iari-7D	7D	179	AX-94993081	AX-94390614	3.3331	8.9242	−0.3057	176.5	179.5
GBaC	T	QGBaC.iari-3A	3A	214	AX-94524087	AX-94567027	4.1584	20.7924	0.3716	212.5	215.5
GNiC	C	QGNiC.iari-4B	4B	147	AX-94623317	AX-94535311	3.3505	12.1994	0.0203	144.5	147.5
GNiC	C	QGNiC.iari-5A	5A	227	AX-95187011	AX-94408752	3.8425	14.4803	−0.0219	221.5	227.5

GCaC, grain calcium concentration; GKC, grain potassium concentration; GFeC, grain iron concentration; GZnC, grain zinc concentration; GMnC, grain manganese concentration; GMoC, grain molybdenum concentration; GCuC, grain copper concentration; GBaC, grain barium concentration; GNiC, grain nickel concentration.

SNPs were located in the gene-rich region of the genome when they were analyzed using BLAST against the reference genome of wheat (*T. aestivum*) from the IWGSC (Ensemble Plants). These chromosomal areas were subjected to *in silico* analysis, which revealed probable candidate genes encoding 30 distinct protein types involved in numerous vital physiological or biochemical processes. SNPs were discovered close to the genes that code for proteins such as glycerol kinase-like, ABC transporter C family, photosynthetic NDH subunit of subcomplex B 3, chloroplastic-like, and aquaporin PIP2-1-like (**Table 4**).

**Table 4:** Putative candidate genes associated with mapped QTL region and gene-encoding proteins.

QTL name	Chr	Transcription ID	Position	UniProt ID	Protein
QGCaC.iari-2A	2A	TraesCS2A02G538000.1	2A: 751,211,771–751,214,150	A0A1D5TDY2	Exocyst complex component EXO70E2-like
QGCaC.iari-2A	2A	TraesCS2A02G515700.1	2A: 739,709,710–739,713,978	A0A1D5TEU4	Glycerol kinase-like
QGKC.iari-6B	6B	TraesCS6B02G041200.1	6B: 25,554,365–25,558,336	A0A1D6SAA1	Subtilisin-like protease SBT3.8
QGFeC.iari-1A.1	1A	TraesCS1A02G205400.1	1A: 367,874,524–367,877,340	A0A1D5RW00	Putative magnesium transporter MRS2-G
QGZnC.iari-2B	2B	TraesCS2B02G133300.2	2B: 99,759,282–99,767,765	A0A1D5UDI0	Probable histone-arginine methyltransferase CARM1
QGZnC.iari-7A	7A	TraesCS7A02G149200.1	7A: 102,525,024–102,537,774	A0A1D6BT82	ABC transporter C family
QGZnC.iari-3B	3B	TraesCS3B02G067400.1	3B: 39,905,297–39,910,826	A0A1D5VKW9	Flocculation protein FLO11-like
QGZnC.iari-7A	7A	TraesCS7A02G470700.2	7A: 666,807,128–666,812,194	A0A1D6BPL1	Protein REVEILLE 6-like isoform X2
QGZnC.iari-3B	3B	TraesCS3B02G094700.1	3B: 63,392,218–63,395,735	W5D6V2	Probable 3 beta-hydroxysteroid-Delta (8) and Delta (7)-isomerase
QGMnC.iari-1B	1B	TraesCS1B02G104000.1	1B: 113,816,236–113,817,684	A0A341P0V6	F-box/LRR-repeat protein At3g59190-like
QGMnC.iari-5A	5A	TraesCS5A02G227700.2	5A: 444,262,734–444,268,781	A0A341V0V1	Microtubule-associated protein futsch-like
QGMnC.iari-7A.1	7A	TraesCS7A02G516300.1	7A: 701,360,512–701,361,053	A0A341Y443	Small polypeptide DEVIL 4-like
QGMnC.iari-1B	1B	TraesCS1B02G426000.1	1B: 652,297,679–652,298,808	Q41585	Type-5 thionin-like
QGMnC.iari-7A.1	7A	TraesCS7A02G106800.1	7A: 64,730,062–64,734,983	A0A1D6BWW2	ATP-dependent 6-phosphofructokinase 6
QGMnC.iari-5A	5A	TraesCS5A02G233200.1	5A: 448,602,858–448,607,957	A0A1D5YC48	Probable potassium transporter 17 isoform X1
QGMnC.iari-7A.2	7A	TraesCS7A02G506000.1	7A: 693,815,402–693,816,263	A0A1D6BJ80	Chaperone protein dnaJ 8, chloroplastic-like
QGMoC.iari-1D	1D	TraesCS1D02G350600.1	1D: 436,189,306–436,197,416	A0A1D5SUX2	Respiratory burst oxidase homolog protein A-like
QGMoC.iari-2A	2A	TraesCS2A02G407700.2	2A: 663,327,624–663,330,251	A0A1D5U7W5	Aquaporin PIP2-1-like
QGMoC.iari-7A	7A	TraesCS7A02G464300.2	7A: 660,550,059–660,555,686	A0A341Y318	Metal transporter Nramp3-like isoform X3
QGMoC.iari-7D	7D	TraesCS7D02G340100.1	7D: 434,910,984–434,912,925	D8L9P6	60S ribosomal protein L37a-1 isoform X2
QGMoC.iari-7D	7D	TraesCS7D02G352700.1	7D: 454,378,164–454,379,979	A0A341Z072	Crocetin glucosyltransferase, chloroplastic-like
QGCuC.iari-4A.1	4A	TraesCS4A02G437600.1	4A: 707,303,998–707,305,576	W5DS07	Disdemethoxycurcumin synthase-like
QGCuC.iari-4A.1	4A	TraesCS4A02G020200.1	4A: 13,757,238–13,759,297	A0A341TIJ0	F-box/kelch-repeat protein At1g55270-like
QGCuC.iari-4A.2	4A	TraesCS4A02G041700.1	4A: 35,181,173–35,183,650	A0A1D5XAR1	Vicilin-like seed storage protein At2g18540
QGBaC.iari-2D	2D	TraesCS2D02G418200.2	2D: 532,176,334–532,185,837	A0A1D5UMB1	Myosin-2 heavy chain-like
QGBaC.iari-4D	4D	TraesCS4D02G285000.1	4D: 455,762,961–455,764,668	W5EG36	50S ribosomal protein L18-like
QGBaC.iari-7B	7B	TraesCS7B02G006600.1	7B: 3,699,820–3,701,864	A0A1D6SE70	Photosynthetic NDH subunit of subcomplex B 3, chloroplastic-like
QGBaC.iari-2D	2D	TraesCS2D02G112800.1	2D: 62,550,720–62,552,140	A0A1D5V1J9	Aspartyl protease family
QGBaC.iari-7B	7B	TraesCS7B02G006800.1	7B: 3,795,056–3,797,564	W5HIH8	Transcription elongation factor 1 homolog
QGNiC.iari-5A	5A	TraesCS5A02G344200.1	5A: 548,625,190–548,627,017	A0A1D5YCZ0	Disease resistance protein RGA5-like

QTL, quantitative trait locus.

### QTL mapping and identification of candidate genes for GCaC, GKC, GFeC, and GZnC

QTL analysis for GCaC revealed one SNP on chromosome 2A with a LOD score of 3.24 and PVE of 14.13% under the control condition. QGCaC.iari-2A encodes the candidate gene (TraesCS2A02G538000.1) exocyst complex component EXO70E2-like and glycerol kinase-like. Two QTLs with LOD scores of 3.53 and 3.89 were identified for GKC under control (QGKC.iari-5B) and NT (QGKC.iari-6B) conditions, respectively (**Table 3**). Transcription ID TraesCS6B02G041200.1 encodes candidate gene subtilisin-like protease SBT3.8 under the NT condition. QTLs such as QGFeC.iari-1A.1 and QGFeC.iari-1A.2 associated with GFeC were mapped on chromosome 1A with LOD scores of 9.31 and 13.42, respectively. TraesCS1A02G205400.1 encodes putative magnesium transporter MRS2-G for GFeC. Furthermore, four QTLs—QGZnC.iari-7A, QGZnC.iari-2B, QGZnC.iari-3B.1, and QGZnC.iari-3B.2—with LOD scores ranging from 3.25 to 6.66 and PVE of 5.29% to 15.13% were mapped for GZnC under control and NT conditions, respectively. TraesCS2B02G133300.2 and TraesCS7A02G149200.1 encode the putative candidate genes probable histone-arginine methyltransferase CARM1 and ABC transporter C family, respectively (**Table 4**).

### QTL mapping and identification of candidate genes for GMnC, GMoC, and GCuC

Four QTLs were identified for GMnC with LOD scores ranging from 3.04 to 3.56 and PVE ranging from 1.1% to 27.83%. Under the control condition, two QTLs (QGMnC.iari-1B and QGMnC.iari-7A.1) were mapped on chromosomes 1B and 7A, respectively and two QTLs (QGMnC.iari-5A and QGMnC.iari-7A.2) were under the NT condition (**Table 3**). TraesCS1B02G104000.1, TraesCS5A02G227700.2, and TraesCS7A02G106800.1 encode F-box/LRR-repeat protein, microtubule-associated protein futsch-like, and ATP-dependent 6-phosphofructokinase, respectively. Moreover, for GMoC, three QTLs under control were mapped, while one QTL was in the NT condition with a LOD score ranging from 3.45 to 6.75. QTLs under the control condition (QGMoC.iari-1D, QGMoC.iari-2A, and QGMoC.iari-7A) of putative candidate gene TraesCS1D02G350600.1, TraesCS7A02G464300.2, and TraesCS7D02G352700.1 code for respiratory burst oxidase homolog protein A-like, metal transporter Nramp3-like isoform X3, and crocetin glucosyltransferase, respectively. Furthermore, two QTLs (QGCuC.iari-4A and QGCuC.iari-4A.2) were mapped for GCuC under the NT condition with LOD scores of 3.31 and 3.41. TraesCS4A02G041700.1 and TraesCS4A02G020200.1 encode vicilin-like seed storage protein and F-box/kelch-repeat protein, respectively (**Table 4**).

### QTL mapping and identification of candidate genes for GBaC and GNiC

Five QTLs in all were mapped for GBaC with LOD scores ranging from 3.07 to 4.15 and PVE ranging from 8.01% to 20.79%. QGBaC.iari-7B, QGBaC.iari-2D, QGBaC.iari-4D, and QGBaC.iari-7D were identified on chromosomes 7B, 2D, 4D, and 7D, respectively, under the control condition, while QGBaC.iari-3A was mapped on chromosome 3A in the NT condition (**Table 3**). In addition, TraesCS4D02G285000.1, TraesCS2D02G112800.1, and TraesCS7B02G006800.1 encode 50S ribosomal protein L18-like, aspartyl protease family protein 2-like, and transcription elongation factor 1 homolog, respectively. Moreover, two QTLs—QGNiC.iari-5A and QGNiC.iari-4B—were mapped for GNiC under the control condition with LOD scores of 3.35 and 3.84. QGNiC.iari-5A harbors one candidate gene; TraesCS5A02G344200.1 encodes disease resistance protein RGA5-like (**Table 4**).

### Selection of superior genotypes

**Figure 4:**
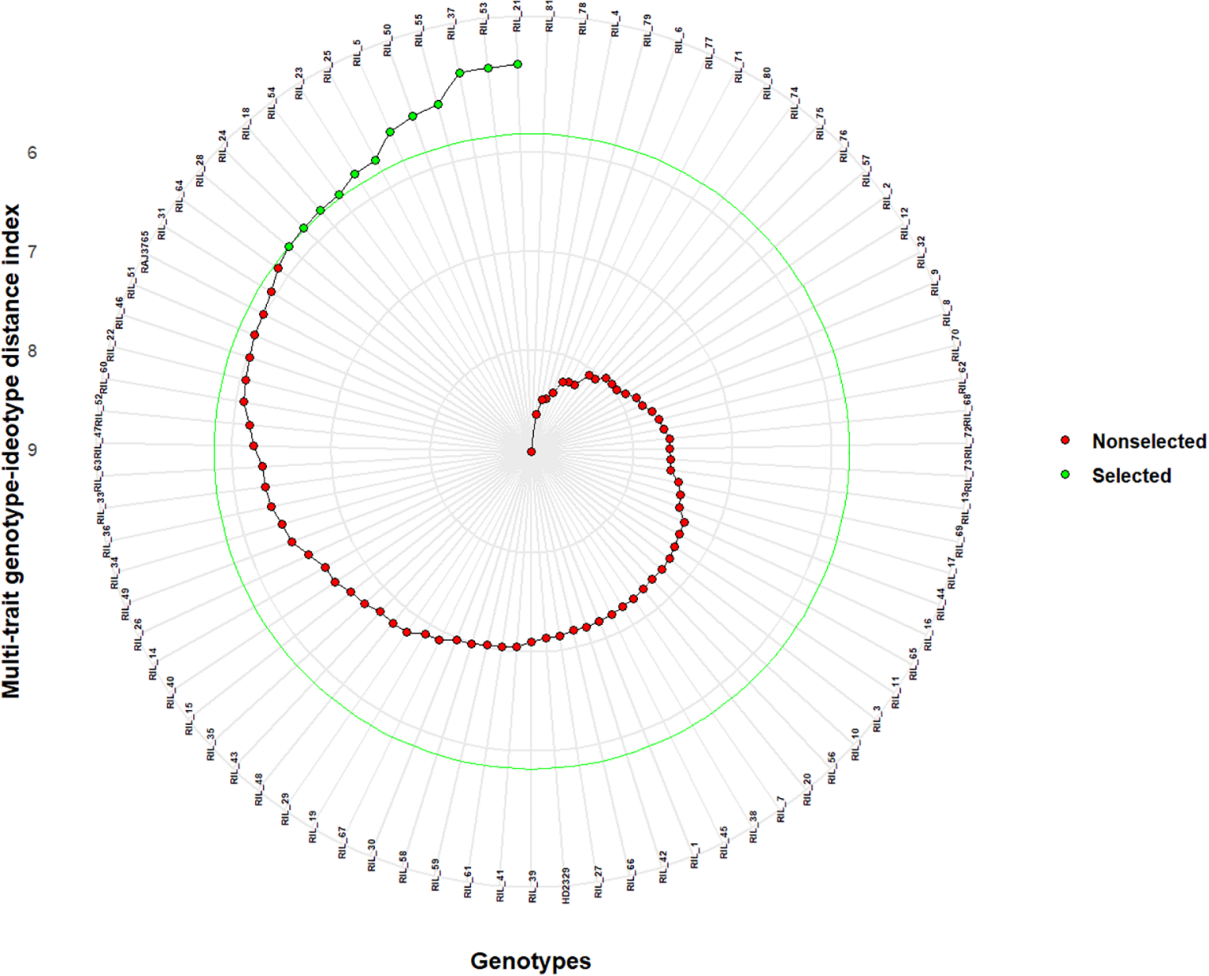
Selection of superior genotypes through multi-trait genotype–ideotype distance index (MGIDI) by applying a 15% selection intensity. The selected recombinant inbred lines (RILs) according to this index are shown in green. Non-selected RILs are represented as red. The central green circle indicates the cut point based on selection intensity.

**Figure 5:**
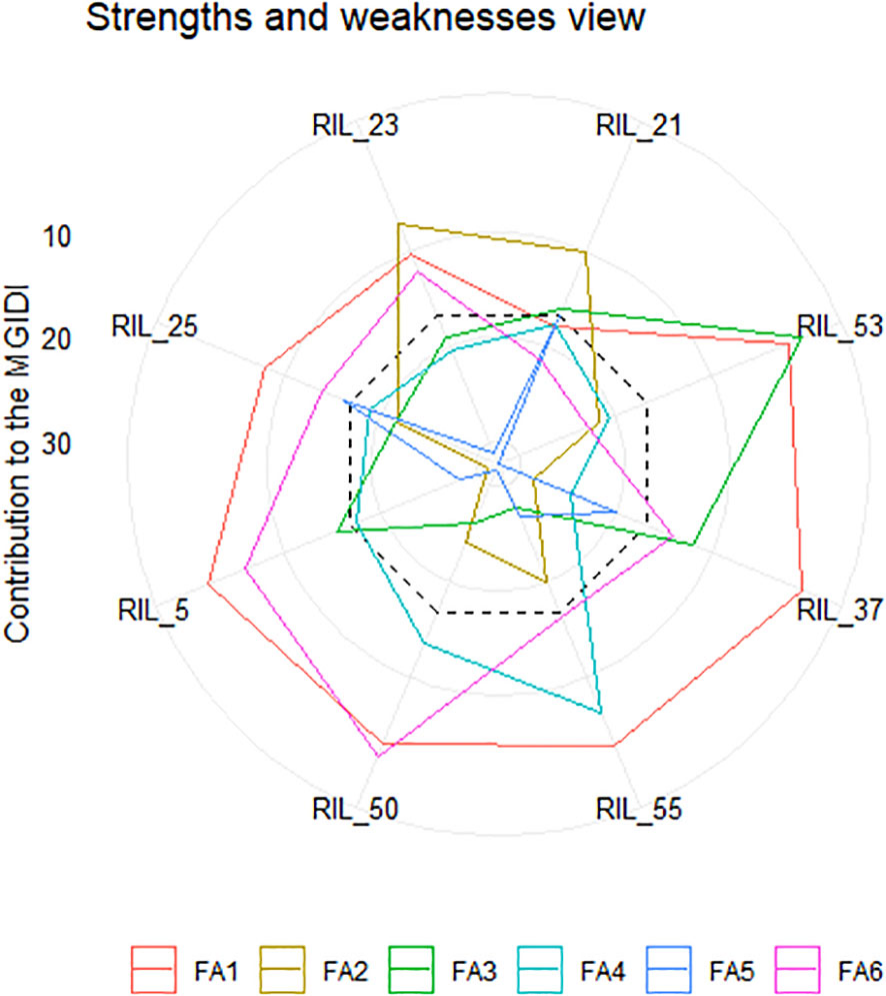
Strength and weakness plot displaying the selected recombinant inbred lines (RILs) represented as the proportion of every factor on the calculated MGIDI. The factors that contributed highly are plotted near the center, while factors that were plotted toward the border had lower contributions. The dashed line represents the theoretical value, assuming equal contributions from each of the factors.

The RILS containing the least MGIDI values were selected as superior genotypes, and the best genotypes were chosen by applying a 15% selection intensity (**Figure 4**). Selected genotypes were based on multiple traits simultaneously. This comprehensive approach ensures a more accurate selection of superior genotypes. Out of parents and their 81 RILs, 12 RILs with high grain nutrient content were selected with 15% selection intensity for further research. Factors that contributed to the MGIDI were categorized into two: low- and high-contribution factors. Factors that had high contributions were placed near the center, whereas factors that had low contributions were placed toward the border. Strength and weakness plot for genotypes showed that FA5 elements (GCuC, GMnC, and GMoC) contributed more and that FA1 elements (GFeC and GNiC) contributed less for the selection of genotypes (**Figure 5**). Furthermore, information about factors associated with correlated elements, selection differential (S), and indicators is given in (**Table 5**).

**Table 5:** Factors associated with different correlated nutrients, selection differential (S), and indicators.

Variables	Factor	Xo	Xs	S	S %	Indicators	Goal
GFeC	FA1	47	49.6	2.53	5.38	Increase	100
GNiC	FA1	0.197	0.217	0.0206	10.5	Increase	100
GCaC	FA2	591	624	32.5	5.49	Increase	100
GKC	FA2	2,931	2,982	50.6	1.73	Increase	100
GMgC	FA2	825	859	34.5	4.19	Increase	100
GAlC	FA3	2.42	2.54	0.113	4.67	Increase	100
GZnC	FA3	33.9	35.8	1.86	5.48	Increase	100
GNaC	FA4	276	279	3.75	1.36	Increase	100
GSrC	FA4	3.88	4.21	0.333	8.59	Increase	100
GCuC	FA5	3.55	3.76	0.21	5.93	Increase	100
GMnC	FA5	12.7	12.5	−0.13	−1.03	Increase	100
GMoC	FA5	0.424	0.594	0.17	40	Increase	100
GBaC	FA6	1.7	1.75	0.0526	3.09	Increase	100

## Discussion

This study reflects the effect of available nitrogen on grain nutrient levels. We found that most of the nutrient concentrations in the control condition were significantly higher than in the nitrogen stress condition; similar findings were also reported in a study by Velu et al. (2016). Similarly, increasing fertilizer use efficiency and improving the yield and quality of cereal grains depend on controlling the use of nitrogen fertilizer doses and the way that nitrogen is supplied to plant vegetation (Ralcewicz et al., 2009; Ercoli et al., 2013; Wojtkowiak et al., 2014). Furthermore, we found a positive association between nitrogen applications on wheat grain nutrient concentration and a similarly positive correlation between nitrogen fertilizers and wheat grain micronutrient content (Kimball et al., 2001; Yue et al., 2007). Additionally, the source and sink relationship of photosynthates and nitrogen can influence the content of micronutrients and proteins in grains (Zhang et al., 2012). We observed a positive correlation between GZnC and GFeC under both control and NT conditions. The foliar application of Zn at the grain-filling stage significantly enhances the grain Fe content; it may be due to Zn-induced Fe translocation from shoots to grains (Ram et al., 2024). GMgC showed a positive correlation with GFeC under control and NT stress conditions. Furthermore, magnesium is involved in nitrogen uptake and controls the photosynthesis process and assimilate distribution between the plant parts (Gastal and Lemaire, 2002; Rubio et al., 2003; Cakmak and Kirkby, 2008).

To manipulate these nutrients, it is necessary to identify genotypes with high-grain nutrient levels and understand the genetic basis of their concentration (Tiwari et al., 2009). In this investigation, we identified a total of 26 QTLs on 17 different chromosomes. Out of 26 QTLs, 18 were found under the control condition, which were associated with GCaC, GKC, GFeC, GZnC, GMnC, GMoC, GBaC, and GNiC. In the case of the NT stress condition, eight QTLs were identified to be linked to GKC, GZnC, GMnC, GMoC, GCuC, and GBaC (**Table 3**). We mapped two QTLs for GKC on chromosomes 5B and 6B; similarly, 34 QTLs were reported in different potassium applications identified on chromosomes 1A, 4A, 6A 1B, 3B, 5B, 7B, and 1D (Zhao et al., 2014). QTL mapping revealed a major QTL for GFeC on chromosome 1A, with the highest LOD score of 13.42 under the control condition, suggesting a crucial role in regulating grain iron accumulation. Two QTLs linked with GFeC were mapped on chromosome 1A, and a linkage map of the population was utilized to map QTL for grain Fe and Zn contents. Following the QTL analysis, two QTLs for grain Fe were found on chromosomes 2A and 7A, whereas one QTL for grain Zn was found on 7A (Tiwari et al., 2009). Additionally, in a *Triticum* sp*elta* and *T. aestivum* RIL population, five QTLs were identified for GFeC, with three QTLs reported on chromosome 1A and two on chromosomes 2A and 3B (Srinivasa et al., 2014). Peleg et al. (2009) mapped five QTLs on chromosomes 2A, 5A, 7A 3B, and 6B for Fe content in tetraploid wild emmer and durum wheat RIL population. QTLs for GZn and GFe have also been found in many chromosomes of wheat and wheat-related species, including 1A, 2A, 2B, 3A, 3D, 4B, 5A, 6A, 6B, 7A, and 7B, with PVE ranging from 2.3% to 14.4% (Krishnappa et al., 2017). The putative candidate gene TraesCS1A02G205400.1 encodes magnesium transporter MRS2-G and regulates the selective Mg21 influx channel within mitochondria by an inbuilt negative feedback process (Schindl et al., 2007). We mapped two QTLs (QGZnC.iari-3B.1 and QGZnC.iari-3B.2) on chromosomes 3B and one QTL each on 2B and 7A for GZnC. We identified one QTL for GZnC on chromosome 3B under both control (AX-94759710) and NT stress (AX-94447080) conditions. Crespo-Herrera et al. (2017) previously identified a QTL for Zn concentration in a population of hexaploid wheat RILs at a similar position on chromosome 3B. Similarly, 40 MTAs were reported in the whole population on chromosomes 2A, 3A, 4A, 5A, 7A, 3B, 5B, 7B, 4D, 5D, 6D, and 7D, whereas chromosomes 3B and 5A had major effects on Zn absorption and transport (Alomari et al., 2018). Stable meta-QTLs indicated that candidate genes like TraesCS2A02G141400, TraesCS3B02G040900, TraesCS4D02G323700, TraesCS3B02G077100, and TraesCS4D02G290900 had effects on micronutrient contents, yield, and yield-related traits (Shariatipour et al., 2021). TraesCS2B02G133300.2 encodes the putative candidate genes probable histone-arginine methyltransferase CARM1, activates the ABA biosynthesis genes OsNCED3 and OsNCED5, and also controls particular methylation at H3K36 (Chen et al., 2021). TraesCS7A02G149200.1 encodes ABC transporter C family members to carry out several tasks, such as ion-channel control, detoxification, heavy metal sequestration, and chlorophyll catabolite transport (Wanke and Uner Kolukisaoglu, 2010). Furthermore, we found two QTLs (QGCuC.iari-4A and QGCuC.iari-4A.2) linked to GCuC under the NT condition, whereas we did not find any QTLs under the control condition. The chromosomal region associated with Xgwm95 on chromosome 2A was the major locus regulating Cu concentration in grain and also maintaining grain Fe, Zn, Mg, and Cu contents in a durum wheat × wild emmer wheat RIL population (Peleg et al., 2009). For GMnC, we found four QTLs on chromosomes 1B, 7A, 5B, and 7A; similarly, Mn co-located with a substantial SNP linked to Mn and Zn on chromosome 1B (Wang et al., 2021). The highest PVE% values of 27.83% and 12.52% were observed on chromosome 7A under both control and NT conditions, respectively. Two QTLs (AX-94535709 and AX-94640059) present on chromosome 7A may be involved in Mn transport or accumulation in wheat grains. TraesCS5A02G233200.1 encodes probable potassium transporter that regulates osmotic potential, salt tolerance, plant growth and development, and also potassium uptake and transport process (Li et al., 2018). Moreover, for GMoC, we mapped four QTLs on chromosomes 1D, 2A, 7A, and 7D. In previous studies, an Mo QTL such as *qMo8* was identified on chromosome 8, which was frequently identified in three field locations and mapped in the same genomic region of rice (Norton et al., 2012; Zhang et al., 2014). The putative candidate gene TraesCS7A02G464300.2 encodes metal transporter Nramp3-like to influence the availability of Fe and Mn, which can indirectly affect Mo uptake and utilization (Agorio et al., 2017). Five QTLs were identified for GBaC on chromosomes 3A, 7B, 2D, 4D, and 7D, which are novel QTLs identified in wheat.

It is essential to save time and resources when testing plant breeding. We used the MGIDI proposed by Olivoto and Nardino (2021) in selecting new donors with high-performance RILs under control and NT stress conditions for further study. A total of 12 wheat RILs (RIL_21, RIL_53, RIL_37, RIL_55, RIL_50, RIL_5, RIL_25, RIL_23, RIL_54, RIL_18, RIL_24, and RIL_28) were identified as elite nitrogen use efficient (NUE) donors based on the MGIDI with 15% selection intensity and the RILs, which were performing even better than the parents (**Figure 4**). The MGIDI view on strengths and weaknesses (**Figure 5**) makes it simple to identify the positive and negative aspects of RILs based on a multiple-trait framework. The strength and weakness plot for genotypes showed that FA5 elements (GCuC, GMnC, and GMoC) contributed more and that FA1 elements (GFeC and GNiC) contributed less to the selection of genotypes. Gabriel et al. (2019) and Olivoto and Nardino (2021) also reported similar findings. Compared to RAJ3765, 14 RILs performed better, and others showed a lower performance, whereas 42 RILs showed higher performance than HD2329. RAJ3765 is the donor/dominant parent that had a higher contribution to superior RIL selection compared to HD2329. RILs showed the highest GFeC of 71.53 mg/kg, while RAJ3765 was 57.77 mg/kg and HD2329 was 64.98 mg/kg under the control condition. In the case of the NT condition, GFeC values were 56.86, 46.21, and 44.69 mg/kg in RILs, RAJ3765, and HD2329, respectively, and a similar trend was observed in GZnC (**Table 2**; **Supplementary Tables S2A, B**). Furthermore, identified QTLs may be used for marker-assisted breeding base varietal development programs or targeted introgression to develop biofortified cultivars for genes linked to nitrogen deficiency in wheat. Identifying a novel source of variation through allele mining efforts in genetic resource collections and putative candidate genes needs to be validated in order to clarify their functional roles in grain nutrient concentration.

## Conclusion

Nitrogen deficit considerably reduces the accumulation of nutrients; in other words, the plant’s nitrogen status is positively associated with every element. ANOVA and descriptive statistics showed a significant difference across all the nutrients. The initial four principal components explained a significant percentage of the total variance, indicating that these traits are crucial in characterizing the genotypes’ responses to different conditions. QTL analysis identified a total of 26 QTLs on 17 distinct chromosomes. Out of 26 QTLs, we mapped 18 QTLs in the control condition and eight QTLs under the nitrogen-deficit condition. We mapped one QTL for GCaC, whereas we mapped two QTLs each for GKC, GFeC, GCuC, and GNiC. We mapped four QTLs each for GZnC, GMnC, and GMoC, while the highest of the five was linked to GBaC. *In silico* analysis of these chromosomal regions revealed putative candidate gene magnesium transporter MRS2-G linked to GFeC and probable histone-arginine methyltransferase CARM1 and ABC transporter C family, which were found to be linked to GZnC. Once these markers are successfully validated, these QTLs can be employed in practical plant breeding for developing biofortified cultivars.

## Data Availability

The datasets presented in this study can be found in online repositories. The names of the repository/repositories and accession number(s) can be found in the article/**Supplementary Material**.
