# Supplementary material for: Mapping of the QTLs governing grain nutrients in wheat (Triticum aestivum L.) under nitrogen treatment using high-density SNP markers
Source: Front Plant Sci. 2025 May 12;16:1553525. doi: 10.3389/fpls.2025.1553525 (PMC12104049; doi:10.3389/fpls.2025.1553525)
Supplement: Supplementary file 1 [file DataSheet1.docx]

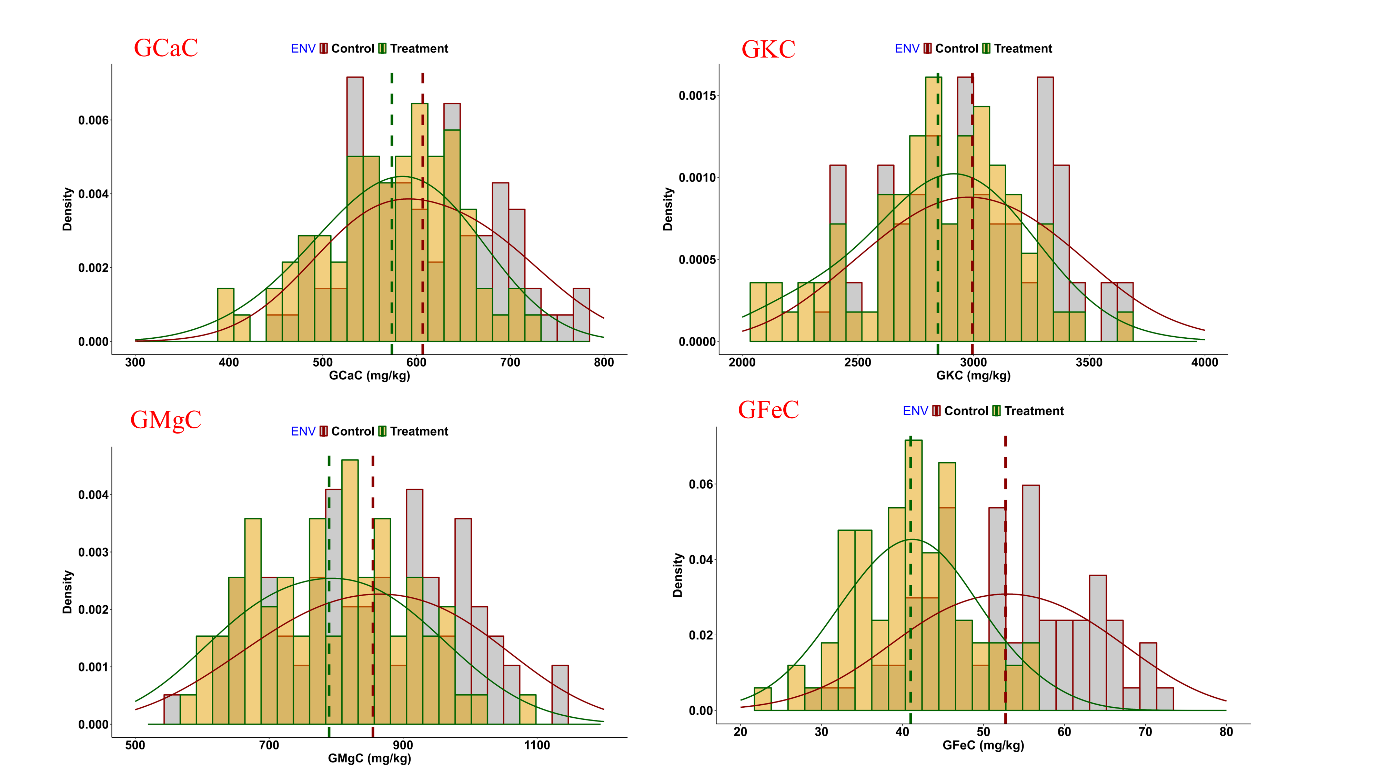


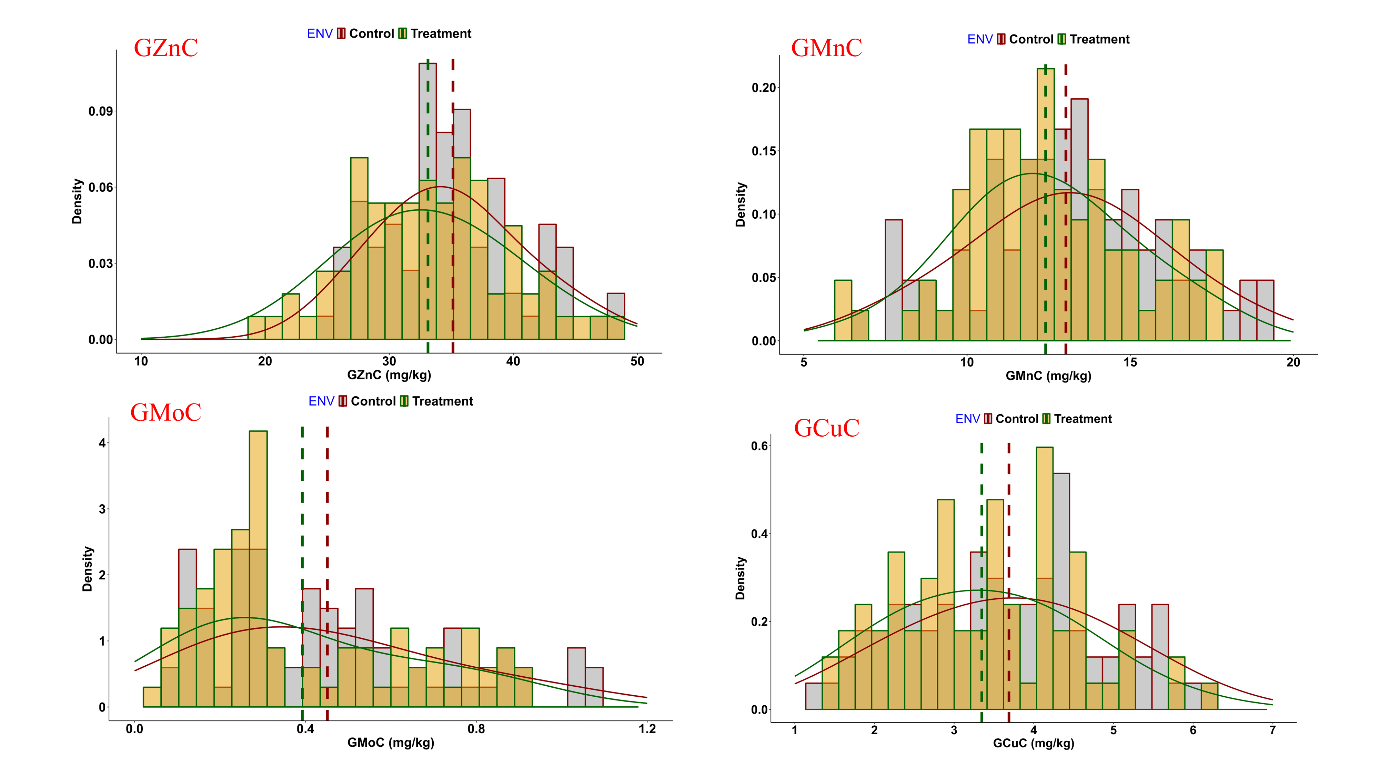


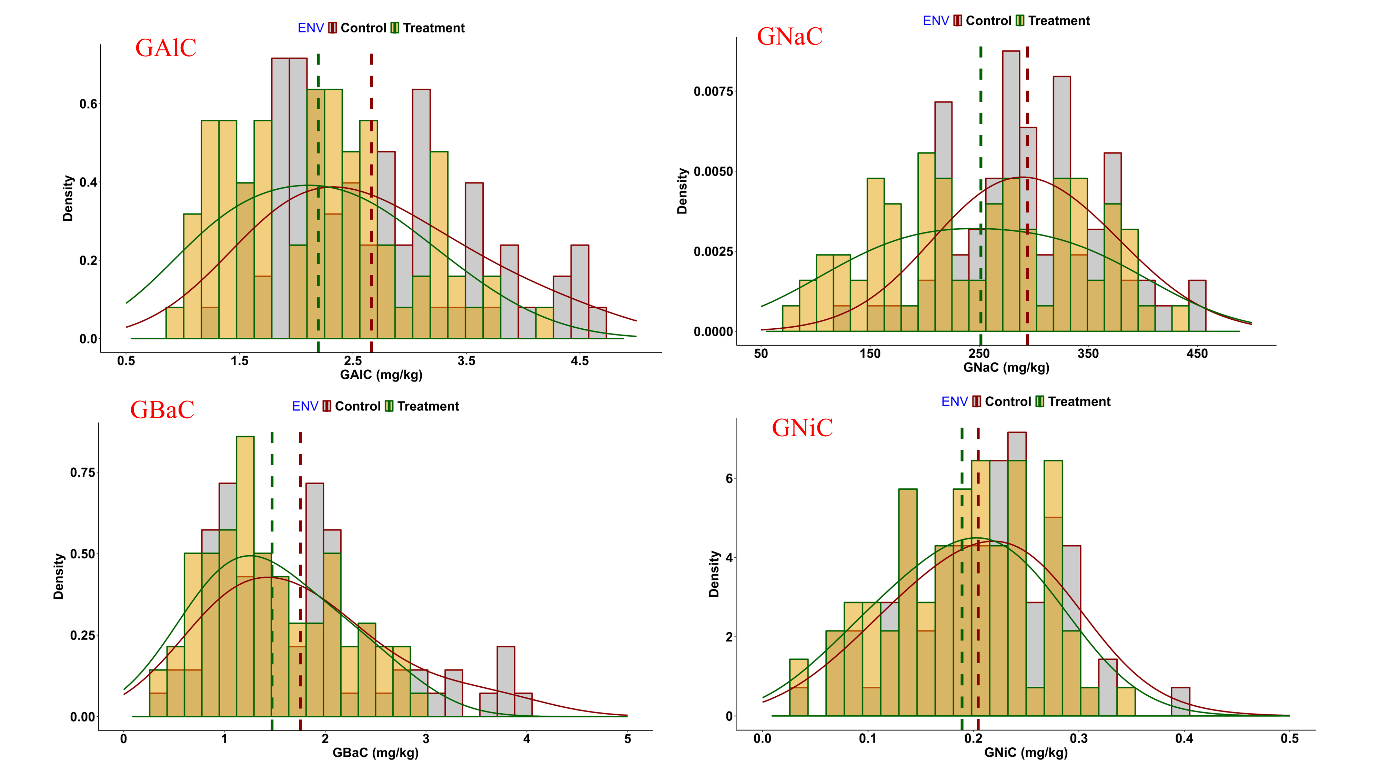


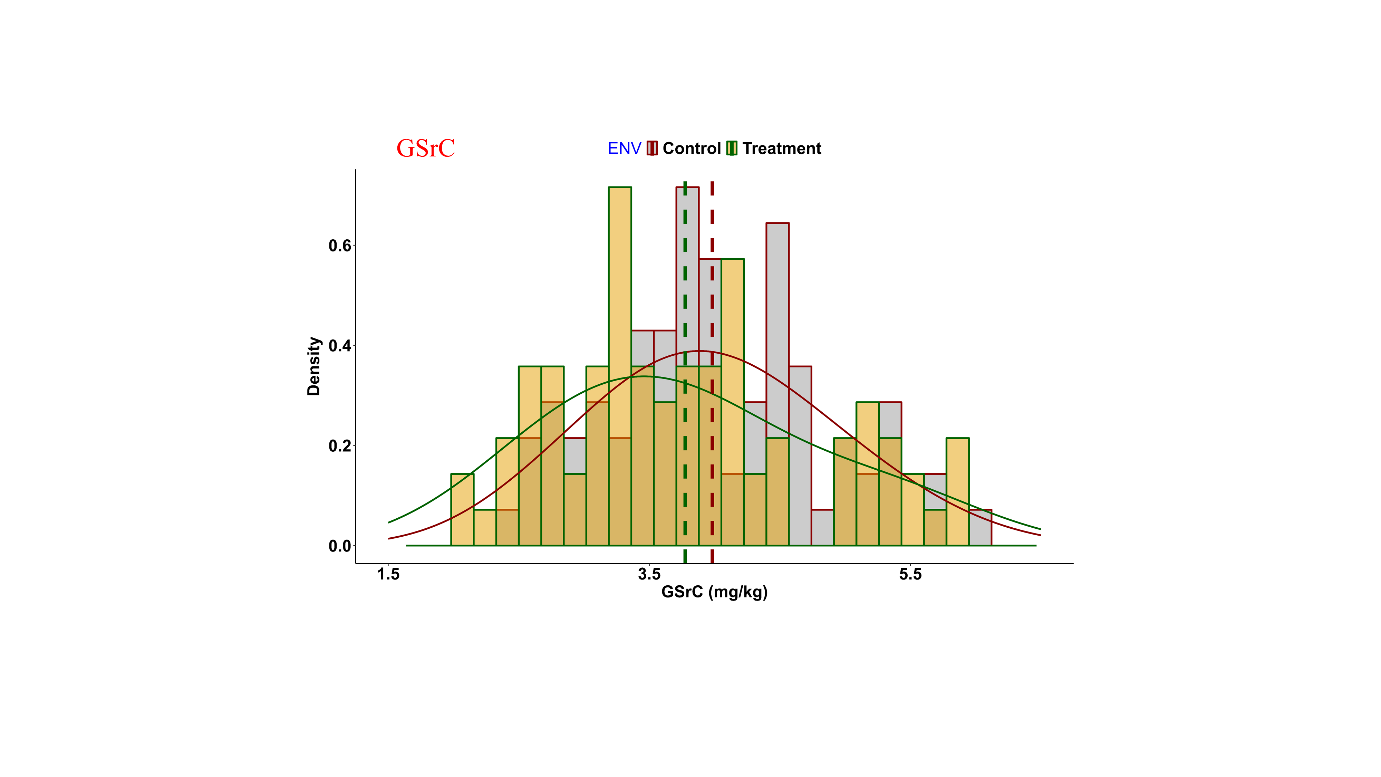


**Supplementary Figure 1**- Trait wise frequency distribution plots of all the studied nutrients in both the environments.


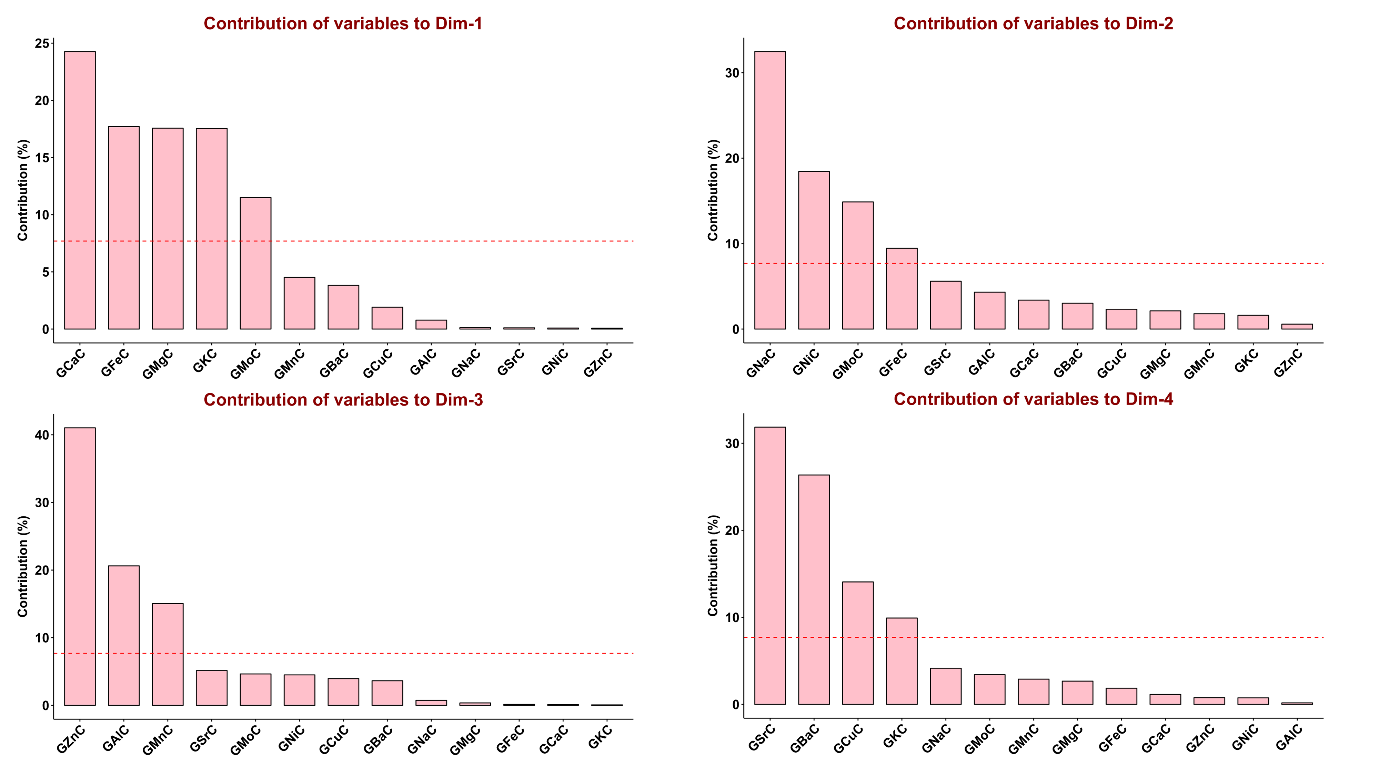


**Supplementary Figure 2A-** Contributions of variables to PCs under control condition


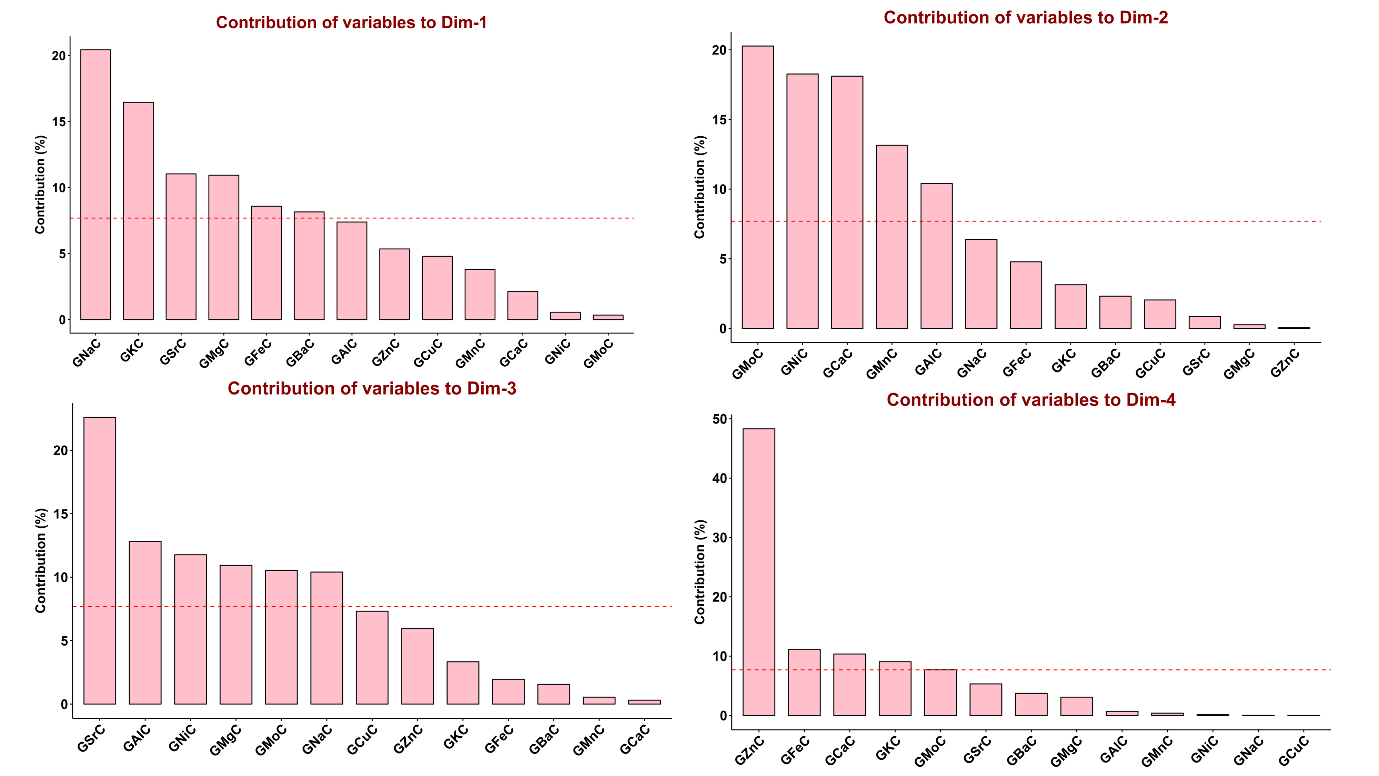


**Supplementary Figure 2B-** Contributions of variables to PCs under nitrogen-deficient (NT) condition

**Supplementary Table 1A- Mean squares and Effect size of nutrient content in grains under control condition**

| Conditions | Traits | Mean Sq | F value | Pr(>F) | Effect Size | Conf. Interval |
| --- | --- | --- | --- | --- | --- | --- |
| Control | GCaC | 12177 | 1.658 | 0.0122* | 0.620 | 591.356-621.990 |
|  | GKC | 249502 | 2.967 | 9.84E-07*** | 0.745 | 2931.679- 3058.218 |
|  | GMgC | 35280 | 3.09 | 4.19E-07*** | 0.753 | 831.208- 878.549 |
|  | GFeC | 199.57 | 22.77 | <2e-16*** | 0.957 | 51.224- 54.382 |
|  | GZnC | 63.71 | 2.322 | 1.00E-04*** | 0.696 | 34.090- 36.183 |
|  | GMnC | 19.364 | 3.505 | 2.55E-08*** | 0.775 | 12.406- 13.499 |
|  | GMoC | 0.16269 | 5.866 | 3.59E-14*** | 0.852 | 0.408-0.504 |
|  | GCuC | 5.636 | 1.659 | 0.0121* | 0.620 | 3.482- 4.141 |
|  | GAlC | 1.4102 | 3.134 | 3.09E-07*** | 0.755 | 2.513- 2.812 |
|  | GNaC | 8861 | 2.825 | 2.67E-06*** | 0.736 | 282.272- 306.271 |
|  | GBaC | 2.4015 | 2.534 | 2.15E-05*** | 0.714 | 1.636- 2.037 |
|  | GNiC | 0.010138 | 4.34 | 1.39E-10*** | 0.810 | 0.192-0.216 |
|  | GSrC | 1.3842 | 2.987 | 8.53E-07*** | 0.746 | 3.839-4.136 |

*** = significant at 0.001% level, * = significant at 0.05% level

**Supplementary Table 1B- Mean squares and Effect size of nutrient content in grains under treatment**

| Conditions | Traits | Mean Sq | F value | Pr(>F) | Effect Size | Conf. Interval |
| --- | --- | --- | --- | --- | --- | --- |
| Treatment | GCaC | 10634 | 1.542 | 0.027* | 0.609 | 558.745-587.818 |
|  | GKC | 217565 | 2.891 | 1.76E-06*** | 0.745 | 2790.676-2909.592 |
|  | GMgC | 27538 | 1.864 | 0.00288** | 0.653 | 767.946-813.123 |
|  | GFeC | 100.19 | 24.66 | <2e-16*** | 0.961 | 39.880-42.126 |
|  | GZnC | 74.04 | 2.916 | 1.48E-06*** | 0.747 | 31.936-34.128 |
|  | GMnC | 13.354 | 2.667 | 8.60E-06*** | 0.729 | 11.924-12.866 |
|  | GMoC | 0.13776 | 14.7 | <2e-16*** | 0.937 | 0.356-0.440 |
|  | GCuC | 2.5083 | 3.703 | 7.70E-09*** | 0.789 | 3.145-3.537 |
|  | GAlC | 1.1715 | 2.87 | 2.04E-06*** | 0.743 | 2.058-2.335 |
|  | GNaC | 15856 | 5.65 | 1.26E-13*** | 0.851 | 236.794-266.835 |
|  | GBaC | 1.518 | 1.336 | 0.0183* | 0.574 | 1.405-1.762 |
|  | GNiC | 0.009412 | 3.58 | 1.70E-08*** | 0.783 | 0.177-0.201 |
|  | GSrC | 2.3526 | 4.775 | 1.29E-11*** | 0.828 | 3.634-4.005 |

*** = significant at 0.001% level, **= significant at 0.01% level * = significant at 0.05% level

**Supplementary Table 2A-** Grain elemental concentration (mg/kg) for RAJ3765 in control and nitrogen deficiency condition

| Trait | Treatment | **Mean** | **Std. Error of Mean** | **SD** | **CV** | **Minimum** | **Maximum** |
| --- | --- | --- | --- | --- | --- | --- | --- |
| GCaC | C | 681.260 | 2.410 | 3.408 | 0.005 | 678.850 | 683.670 |
|  | T | 710.130 | 1.460 | 2.065 | 0.003 | 708.670 | 711.590 |
| GKC | C | 3609.415 | 136.915 | 193.627 | 0.054 | 3472.500 | 3746.330 |
|  | T | 3400.395 | 242.625 | 343.124 | 0.101 | 3157.770 | 3643.020 |
| GMgC | C | 979.670 | 37.690 | 53.302 | 0.054 | 941.980 | 1017.360 |
|  | T | 929.500 | 74.240 | 104.991 | 0.113 | 855.260 | 1003.740 |
| GFeC | C | 57.325 | 0.445 | 0.629 | 0.011 | 56.880 | 57.770 |
|  | T | 46.030 | 0.180 | 0.255 | 0.006 | 45.850 | 46.210 |
| GZnC | C | 33.265 | 0.665 | 0.940 | 0.028 | 32.600 | 33.930 |
|  | T | 29.745 | 0.795 | 1.124 | 0.038 | 28.950 | 30.540 |
| GMnC | C | 14.250 | 0.380 | 0.537 | 0.038 | 13.870 | 14.630 |
|  | T | 10.305 | 0.625 | 0.884 | 0.086 | 9.680 | 10.930 |
| GMoC | C | 0.370 | 0.010 | 0.014 | 0.038 | 0.360 | 0.380 |
|  | T | 0.230 | 0.020 | 0.028 | 0.123 | 0.210 | 0.250 |
| GCuC | C | 5.240 | 0.400 | 0.566 | 0.108 | 4.840 | 5.640 |
|  | T | 4.410 | 0.270 | 0.382 | 0.087 | 4.140 | 4.680 |
| GAlC | C | 3.790 | 0.190 | 0.269 | 0.071 | 3.600 | 3.980 |
|  | T | 1.400 | 0.020 | 0.028 | 0.020 | 1.380 | 1.420 |
| GNaC | C | 355.295 | 11.375 | 16.087 | 0.045 | 343.920 | 366.670 |
|  | T | 431.270 | 22.130 | 31.297 | 0.073 | 409.140 | 453.400 |
| GBaC | C | 1.180 | 0.030 | 0.042 | 0.036 | 1.150 | 1.210 |
|  | T | 1.155 | 0.075 | 0.106 | 0.092 | 1.080 | 1.230 |
| GNiC | C | 0.285 | 0.005 | 0.007 | 0.025 | 0.280 | 0.290 |
|  | T | 0.135 | 0.005 | 0.007 | 0.052 | 0.130 | 0.140 |
| GSrC | C | 3.495 | 0.045 | 0.064 | 0.018 | 3.450 | 3.540 |
|  | T | 3.200 | 0.030 | 0.042 | 0.013 | 3.170 | 3.230 |

**Supplementary Table 2B.** Grain elemental concentration (mg/kg) for HD2329 in control and nitrogen deficiency condition

| Trait | Treatment | **Mean** | **Std. Error of Mean** | **SD** | **CV** | **Minimum** | **Maximum** |
| --- | --- | --- | --- | --- | --- | --- | --- |
| GCaC | C | 638.640 | 4.000 | 5.657 | 0.009 | 634.640 | 642.640 |
|  | T | 571.770 | 23.980 | 33.913 | 0.059 | 547.790 | 595.750 |
| GKC | C | 2851.040 | 385.300 | 544.896 | 0.191 | 2465.740 | 3236.340 |
|  | T | 3254.895 | 169.245 | 239.349 | 0.074 | 3085.650 | 3424.140 |
| GMgC | C | 841.875 | 4.285 | 6.060 | 0.007 | 837.590 | 846.160 |
|  | T | 881.065 | 49.075 | 69.403 | 0.079 | 831.990 | 930.140 |
| GFeC | C | 64.185 | 0.795 | 1.124 | 0.018 | 63.390 | 64.980 |
|  | T | 44.100 | 0.590 | 0.834 | 0.019 | 43.510 | 44.690 |
| GZnC | C | 24.590 | 0.330 | 0.467 | 0.019 | 24.260 | 24.920 |
|  | T | 26.380 | 2.480 | 3.507 | 0.133 | 23.900 | 28.860 |
| GMnC | C | 13.135 | 1.445 | 2.044 | 0.156 | 11.690 | 14.580 |
|  | T | 9.555 | 0.375 | 0.530 | 0.056 | 9.180 | 9.930 |
| GMoC | C | 0.460 | 0.010 | 0.014 | 0.031 | 0.450 | 0.470 |
|  | T | 0.255 | 0.025 | 0.035 | 0.139 | 0.230 | 0.280 |
| GCuC | C | 7.360 | 0.610 | 0.863 | 0.117 | 6.750 | 7.970 |
|  | T | 2.355 | 0.315 | 0.445 | 0.189 | 2.040 | 2.670 |
| GAlC | C | 2.065 | 0.035 | 0.049 | 0.024 | 2.030 | 2.100 |
|  | T | 1.260 | 0.060 | 0.085 | 0.067 | 1.200 | 1.320 |
| GNaC | C | 378.545 | 4.015 | 5.678 | 0.015 | 374.530 | 382.560 |
|  | T | 348.545 | 20.535 | 29.041 | 0.083 | 328.010 | 369.080 |
| GBaC | C | 1.660 | 0.260 | 0.368 | 0.222 | 1.400 | 1.920 |
|  | T | 1.330 | 0.050 | 0.071 | 0.053 | 1.280 | 1.380 |
| GNiC | C | 0.190 | 0.010 | 0.014 | 0.074 | 0.180 | 0.200 |
|  | T | 0.140 | 0.020 | 0.028 | 0.202 | 0.120 | 0.160 |
| GSrC | C | 4.620 | 0.270 | 0.382 | 0.083 | 4.350 | 4.890 |
|  | T | 3.060 | 0.030 | 0.042 | 0.014 | 3.030 | 3.090 |

**Supplementary Table 3- Eigen value, percentage of variance and cumulative variance under control and treatment conditions.**

| Condition | Components | Eigen value | Percentage of variance | Cumulative variance |
| --- | --- | --- | --- | --- |
| Control | 1 | 2.367629 | 18.21253 | 18.21253 |
|  | 2 | 1.949551 | 14.99655 | 33.20907 |
|  | 3 | 1.524974 | 11.73057 | 44.93965 |
|  | 4 | 1.380863 | 10.62202 | 55.56167 |
|  | 5 | 1.214221 | 9.340159 | 64.90183 |
|  | 6 | 0.881089 | 6.777611 | 71.67944 |
|  | 7 | 0.801261 | 6.163545 | 77.84298 |
|  | 8 | 0.738969 | 5.684377 | 83.52736 |
|  | 9 | 0.594762 | 4.575093 | 88.10245 |
|  | 10 | 0.503789 | 3.875303 | 91.97776 |
|  | 11 | 0.40916 | 3.147382 | 95.12514 |
|  | 12 | 0.368653 | 2.83579 | 97.96093 |
|  | 13 | 0.265079 | 2.039072 | 100 |
| Condition | Components | Eigen value | Percentage of variance | Cumulative variance |
| Treatment | 1 | 2.4430559 | 18.792738 | 18.79274 |
|  | 2 | 1.8111032 | 13.931563 | 32.7243 |
|  | 3 | 1.3600588 | 10.461991 | 43.18629 |
|  | 4 | 1.1940697 | 9.185151 | 52.37144 |
|  | 5 | 1.0647135 | 8.190104 | 60.56155 |
|  | 6 | 0.9500327 | 7.307944 | 67.86949 |
|  | 7 | 0.9381975 | 7.216904 | 75.0864 |
|  | 8 | 0.8918305 | 6.860235 | 81.94663 |
|  | 9 | 0.6218029 | 4.783099 | 86.72973 |
|  | 10 | 0.5663825 | 4.356788 | 91.08652 |
|  | 11 | 0.4513181 | 3.471677 | 94.55819 |
|  | 12 | 0.3872576 | 2.978905 | 97.5371 |
|  | 13 | 0.320177 | 2.4629 | 100 |
